# Breast milk paclitaxel excretion following intravenous chemotherapy—a case report

**DOI:** 10.1038/s41416-019-0529-z

**Published:** 2019-07-31

**Authors:** Christopher G. C. A. Jackson, Tessa Morris, Noelyn Hung, Tak Hung

**Affiliations:** 1 Department of Medicine, Otago Medical School, University of Otago, PO Box 56, Dunedin, 9054 New Zealand; 2 Southern Blood and Cancer Service, Dunedin, New Zealand; 3 Zenith Technology Corporation Limited, Dunedin, New Zealand

**Keywords:** Breast cancer, Chemotherapy

## Abstract

Breast cancer can be diagnosed during pregnancy and in the peri-partum period, and the potential exposure of a foetus or neonate to chemotherapy is of concern to mothers and clinicians. Paclitaxel is a commonly used agent in breast cancer, but little is known about its excretion in breast milk. Breastfeeding during chemotherapy has been traditionally cautioned against due to the risk of neonatal exposure to chemotherapy agents, however, data are limited. We measured serum and breast milk concentrations of paclitaxel in a 33-year-old woman with an early breast cancer diagnosed during pregnancy and treated with weekly paclitaxel 80 mg/m^2^. We found breast milk paclitaxel levels drop below the minimum quantifiable dose at 72 h following chemotherapy, with a relative infant dose of 0.091%. Breast milk excretion of paclitaxel following a dose of 80 mg/m^2^ is negligible at 72 h, and this may be a safe time to recommence breastfeeding following exposure.

## Introduction

Paclitaxel is used in the treatment of a wide range of solid organ malignancies including breast, lung, ovarian, endometrial and upper gastrointestinal cancers.^1^ Paclitaxel is delivered intravenously as it has poor oral bioavailability, limited by *p*-glycoprotein excretion from enterocytes and CYP450 enzyme metabolism. Novel oral paclitaxel formulations are currently being developed.

Breast cancer is one of the most common causes of invasive cancer in pregnant women.^2^ Although only 0.4% of all breast cancers are diagnosed during pregnancy, 20% of breast cancer patients under the age of 30 will be pregnant at the time of diagnosis,^2,3^ and the incidence of cancer diagnoses during pregnancy is rising.^2–4^

Breastfeeding is important for neonatal nutrition, immune and neurocognitive development and maternal bonding.^4,5^ Breastfeeding has traditionally been cautioned against during chemotherapy due to the risk of neonatal exposure to chemotherapy agents,^2,4^ however, there is very limited information available about the excretion of chemotherapeutic agents into human body fluids, including breast milk. As some patients will be diagnosed with breast cancer and require chemotherapy when they are potentially lactating, data are needed to reliably inform patients about potential exposure rather than relying on non-evidence-based assumptions.

Combination chemotherapy regimens for high-risk early breast cancer contain an anthracycline and a taxane.^6,7^ A systematic review of 50 patients receiving taxanes during pregnancy showed adequate pregnancy outcomes,^2,8^ however, data regarding safety in lactating patients is limited, and current guidelines recommend against breastfeeding during any chemotherapy.^9^

Drug excretion into breast milk is related to maternal plasma concentration and half-life, lipid solubility, protein binding, molecular size and ionisation.^4,5,10^ Pharmacokinetic studies have demonstrated fluorouracil is undetectable in breast milk during or after chemotherapy,^4^ however, high breast milk levels of doxorubicin and its active metabolite were found up to 72 h following chemotherapy with increased breast milk concentrations compared to plasma,^4,11^ and cyclophosphamide excretion into breast milk has been demonstrated.^3,4^

Relative infant dose is a measure used to estimate the average daily dosage of drug a breastfed infant would receive based on measured drug concentrations in breast milk and the maternal drug dose.^10^ In general an RID of <5–10% is considered to be acceptable.^10,12^

With regard to taxanes, a case study of a patient receiving intravenous paclitaxel and carboplatin for papillary thyroid cancer (paclitaxel 30 mg/m^2^ and carboplatin AUC 1.5 weekly) showed carboplatin had an RID of 2% and continued to be present in breast milk at 316 h following the final cycle of weekly chemotherapy. Paclitaxel had an RID of 16.7% and was eliminated before 316 h following chemotherapy.^13^

We sought to determine breast milk excretion of paclitaxel by undertaking a case study of a lactating woman receiving weekly paclitaxel chemotherapy at contemporary doses for breast cancer.

## Case

The patient is a 33-year-old gravida four, para two woman who self-detected a left axillary mass whilst 17 weeks pregnant. There was no definite immediate family history of breast or ovarian cancer. BRCA mutation testing was negative. Ultrasound revealed a 3 cm axillary hypervascular hypoechoic mass with microcalcifications. Mammography revealed microcalcifications over 3.6 cm in the left axilla. Staging chest X-ray and abdominal ultrasound were unremarkable. Ultrasound guided biopsy showed a high-grade carcinoma consistent with primary breast origin, oestrogen receptor (ER) and progesterone receptor (PR) negative, and HER-2 negative by fluorescent in situ hybridisation (FISH). Following informed discussion with the patient a left mastectomy was undertaken at 20 weeks’ gestation with the histological findings of a 9 mm grade 3, ER negative, PR negative and HER-2 negative, node-positive (4/17 nodes) invasive ductal carcinoma (pT1b pN2a) of the left breast.

Adjuvant chemotherapy with three cycles of fluorouracil 500 mg/m^2^, epirubicin 100 mg/m^2^ and cyclophosphamide 500 mg/m^2^ (FEC) every 3 weeks, followed by nine cycles of weekly paclitaxel (80 mg/m^2^) was administered, with a shorter course of paclitaxel delivered due to concerns regarding cumulative neurotoxicity. Chemotherapy was paused after six cycles of paclitaxel at 36 weeks’ gestation for Caesarean section delivery at 38 weeks and 6 days, as the patient had undergone two previous Caesarean sections. The infant’s birthweight was 3700 g, with Apgar scores of 9 and 10.

Following delivery, the patient decided to breastfeed after discussion with the clinicians involved in her care, and the baby was bottle fed when chemotherapy recommenced 2 weeks after delivery. As the patient wished to resume breastfeeding following chemotherapy, she used a breast pump to express and discard the breast milk until chemotherapy was completed.

We collected breast milk and plasma samples after the 9th week of weekly paclitaxel to undertake pharmacokinetic analysis and assess breast milk paclitaxel excretion.

Following the completion of chemotherapy, adjuvant radiotherapy was delivered to the chest wall, axilla and supraclavicular fossa to a dose of 50Gray in 25 fractions. Adjuvant hormonal therapy was not recommended as hormone receptor status was negative.

## Method

Maternal plasma samples were collected pre-chemotherapy, and following intravenous paclitaxel at 0.25, 0.50, 0.75, 1.00, 1.25, 1.50, 1.75, 2.00, 2.50, 3.50, 4.50, 6.50 and 8.50 h. All expressed breast milk was collected and frozen from time point zero until 15 days following chemotherapy. All samples of breast milk expressed within 24 h of chemotherapy were analysed, beyond 24 h post-chemotherapy selective analysis of samples was undertaken.

To analyse paclitaxel concentrations in breast milk a standard curve was prepared. We pipetted 200 ul of milk sample into a 1.2 mL 96-well plate, added 50 μL of internal standard docetaxel (6 μg/mL) into each well (except for the blank and subject’s blank) and vortexed the plate for 2 min at 2000 rpm, extracted samples with 0.6 mL tert-butyl methyl ether using Tomtec Quadra 3 and dry extracted solvent under a stream of nitrogen. Triplicate samples were reconstituted with 200 μl acetonitrile:B.P. water (5:95, v/v) containing 0.1% (v/v) formic acid and inject 10 μL of sample into a LC/MS/MS system. The samples were separated with Luna C18 column and detected with a triple-quadrupole mass spectrometer using electrospray ionisation in positive mode and multiple reaction monitoring. The precursor-product ion transition values for paclitaxel m/z 854.5 > 286.0 and 808.4 > 527.2 for the internal standard docetaxel. The ratio of the peak area of paclitaxel to the internal standard docetaxel was used for calibration and measurement of unknown concentrations of paclitaxel.

Maternal plasma sampling was undertaken as described, all samples were analysed for paclitaxel concentration (Table 1). All expressed breast milk was collected and frozen for storage. All breast milk samples within 24 h of chemotherapy were analysed for paclitaxel concentration, collected at 2.75, 6.00, 9.00, 12.00 and 22.50 hours post-dose. Beyond 24 h post-chemotherapy selective analysis of samples at time points 27.00, 71.25, 75.75, 142.25, 146.5, 189.5, 266.25, 313.00, 359.75 h were analysed (Table 1).

**Table 1:** Maternal plasma and breast milk paclitaxel concentrations

Time (h)	Maternal plasma concentration (ng/mL)	Breast milk concentration (ng/mL)
0	0.00	
0.25	855.17	
0.5	1341.83	
0.75	1949.07	
1	2050.92	
1.25	1135.39	
1.5	446.10	
1.75	318.53	
2	227.15	
2.5	164.38	
3	129.90	111.4
3.5	100.88	
4.5	67.23	
6	56.41	63.48
9		36.73
12		23.84
22.5		8.99
27		6.83
71.25		<mqc
75.75		<mqc
142.25		<mqc
146.5		<mqc
189.5		<mqc
193		<mqc
266.25		<mqc
313		<mqc
359.75		<mqc

*mqc* minimum quantifiable concentration (2.5 ng/mL)

Relative infant dose (RID) was calculated using the following formulae,^10^ and a 50th centile estimated infant weight.^14^

$$\hskip 50pt	{\mathrm{Infant}}\,{\mathrm{daily}}\,{\mathrm{dose}}\left( {{\mathrm{mg/kg/day}}} \right)\\ 	= {\mathrm{Drug}}\,{\mathrm{concentration}}\,{\mathrm{in}}\,{\mathrm{milk}}\left( {{\mathrm{mg/mL}}} \right)\\ 	 \hskip 12pt \times {\mathrm{volume}}\,{\mathrm{milk}}\,{\mathrm{consumed}}\,{\mathrm{daily}}\left( {{\mathrm{mL/kg}}} \right)\\ 	\hskip 12pt\ast \left( {{\mathrm{standard}}\,{\mathrm{daily}}\,{\mathrm{milk}}\,{\mathrm{volume}} = {\mathrm{150mL/kg}}} \right)\\ 	 \hskip -30pt{\mathrm{RID}}\left( {\mathrm{\% }} \right) = \frac{{{\mathrm{Infant}}\,{\mathrm{dose}}\left( {{\mathrm{mg/kg/day}}} \right)}}{{{\mathrm{Maternal}}\,{\mathrm{dose}}\left( {{\mathrm{mg/kg/day}}} \right)}} \times 100$$

## Results

**Fig. 1:**
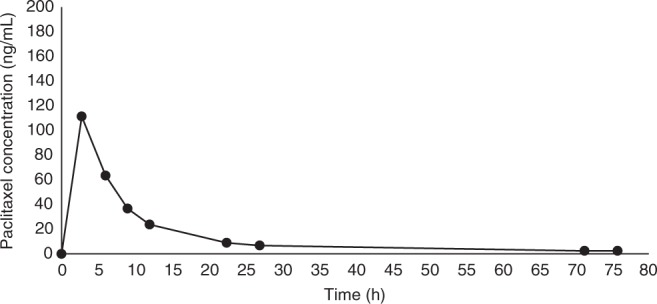
Breast milk paclitaxel concentration graph

**Fig. 2:**
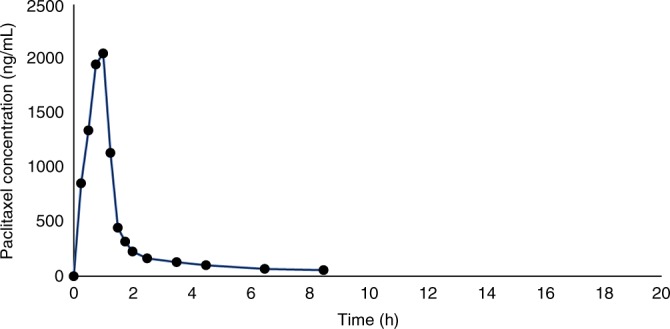
Maternal plasma paclitaxel concentration graph

**Fig. 3:**
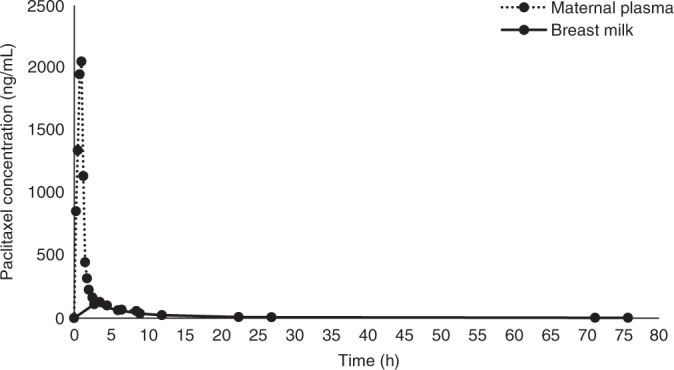
Comparison graph maternal plasma and breast milk paclitaxel concentrations

Peak plasma paclitaxel concentrations of 2050 ng/mL were reached 1 h following chemotherapy, with a rapid decline in plasma concentration over the following seven-and-a-half hours. Peak breast milk paclitaxel concentrations were reached 2.75 h following chemotherapy, and levels below the minimum quantifiable concentration (<2.5 ng/mL) were reached at 71.25 h. Plasma and breast milk paclitaxel concentrations are shown in Figs. 1 and 2. A comparison of plasma and breast milk concentration is shown in Fig. 3.

Based on a breast milk paclitaxel concentration of 6.83 ng/mL at 27 h following chemotherapy, the patient’s post-delivery weight of 63.3 kg and an estimated infant weight of 4.75 kg (50th centile for a 4-week-old baby^14^) RID is 0.247% at 27 h following treatment. At 72 h following chemotherapy the maximum relative infant dose is 0.091%.

## Discussion

Our case demonstrates paclitaxel is excreted into breast milk after intravenous administration, reaching levels below the minimum quantifiable concentrations at 71.25 h following chemotherapy. Based on these results, at 72 h following chemotherapy the maximum a breastfed infant would ingest is 0.091% of the maternal dose.

Adult clinical studies have been undertaken to investigate the oral route of paclitaxel administration. Oral paclitaxel has poor bioavailability, limited by p-glycoprotein excretion from enterocytes and CYP3A4 metabolism.^15,16^ Given the limited absorption of oral paclitaxel it is unlikely breastfed infants will have meaningful systemic exposure to paclitaxel beyond 72 h following chemotherapy, however infant gut transport, absorption, metabolism and excretion processes are immature in comparison to those in adults,^17^ and as a result the impact of low levels of paclitaxel exposure are not yet known.

Given the rising incidence of cancer diagnoses during pregnancy, and as patients generally want to breastfeed whenever possible, information regarding breast milk expression of chemotherapy drugs is required to inform patient decision making.

Our case study will assist clinicians and patients to make informed decisions about breastfeeding during paclitaxel chemotherapy. Further research is needed to investigate chemotherapeutic agent expression in breast milk, and to guide decision making for breastfeeding patients receiving cancer treatment.

## Data Availability

All data generated or analysed during this study are included in this publication.
